# When is synthetic sufficient? Ethical considerations and alternatives in simulation-based ultrasound education

**DOI:** 10.1186/s41077-024-00327-x

**Published:** 2025-01-14

**Authors:** Andrea J. Doyle, Claire M. Condron

**Affiliations:** RCSI SIM Centre for Simulation Education and Research, RCSI University of Medicine and Health Sciences, Dublin, Ireland

**Keywords:** Simulation-Based Education (SBE), Ultrasound training, Replacement, Reduction, Refinement, Live tissue training (LTT), Animal models, EThical considerations

## Abstract

Simulation-based education (SBE) has become an integral part of training in health professions education, offering a safe environment for learners to acquire and refine clinical skills. As a non-ionising imaging modality, ultrasound is a domain of health professions education that is particularly supported by SBE. Central to many simulation programs is the use of animal models, tissues, or body parts to replicate human anatomy and physiology. However, along with its educational benefits, the use of animals in SBE generates a considerable amount of waste, raising important environmental and ethical concerns. Although research indicates that animal models yield comparable educational outcomes to synthetic models, animal models continue to be preferred in surgical and medical training. In response to these challenges, the principles of Replacement, Reduction, and Refinement (the 3Rs) have emerged as guiding standards to minimise the impact of animal use in research and education. Furthermore, synthetic models align with 3R principles, addressing ethical and environmental issues by reducing animal dependence and waste generation. Synthetic models offer key educational benefits over animal models by closely mimicking human anatomy and pathophysiology, providing consistent and anatomically accurate training. Unlike animal models, they eliminate variability in tissue properties, ensuring standardised and reliable experiences. Moreover, synthetic models can simulate specific pathologies, enabling targeted learning that may be difficult with animal tissue. Resistance related to clinical relevance and preference for animal-based SBE is a persisting challenge that might be overcome through the development of clinically and anatomically relevant tissue-mimicking materials, like those previously developed for other applications such as quality assurance phantoms in diagnostic imaging. The involvement of knowledge or end-user engagement, along with evidence-based design solutions, is crucial to catalyse a paradigm shift in a discipline deeply entrenched in tradition. The combined expertise, skills, and perspectives of medical professionals, educators, academic researchers, and industry specialists could collaboratively develop alternative methods to simulate live animal scenarios, replacing and reducing animal tissue dependence in SBE.

## The expansion of ultrasound education in health professions education

Once a specialised skill for sonographers, radiographers, and radiologists, ultrasound has gained importance across a wide array of health professions and medical disciplines with the adoption of point of-care ultrasound (PoCUS) [1]. PoCUS enables immediate, bedside diagnostic information, making it invaluable in diverse specialties, from emergency medicine to primary care. In paediatric emergency medicine, PoCUS enhances the efficiency and effectiveness of procedures, expanding the procedural capabilities of emergency physicians, and improving success rates and safety [2]. In the intensive care unit (ICU), PoCUS aids in diagnostic and therapeutic decision-making for critically ill patients, promoting resource efficiency and potentially reducing the duration of mechanical ventilation [3]. Studies have shown that in primary care, POCUS exhibits high inter-rater agreement and reliability, and can provide crucial clinical information to inform further decision-making using POCUS [4].

The rapid and widespread clinical integration of ultrasound has driven parallel growth in health professions education, highlighting the need for standardised training protocols, opportunities for deliberate practice, and comprehensive competency assessments to ensure safe and effective use [5–7]. Simulation-based education (SBE) has become a fundamental component of ultrasound training, complementing clinical practice by providing learners with a safe environment to develop and refine their skills. However, standardising PoCUS training across institutions presents challenges, including the need to reach a consensus on key areas such as the scope of use, credentialing, documentation, quality assurance, leadership, governance, teaching, research, and equipment maintenance [8, 9]. Despite these challenges, the expansion of ultrasound training emphasises the need for high-quality, realistic training models made from ultrasound-compatible materials. These models enable practitioners to simulate various clinical scenarios in a controlled setting, allowing skill development without risk to patients. To enhance the learning experience, these materials must closely replicate human tissues and organs, providing realistic feedback during scanning.

Simulated participant (SP) methodology is often utilised in ultrasound training, simulating clinical scenarios to develop both technical and communication skills [10]. However, SP engagement can be limited, as specific pathologies cannot be consistently modelled in real individuals. Within this context, animal models, tissues, or body parts have historically been used to replicate human anatomy and provide hands-on practice. While these models offer clinical relevance, they present significant ethical and environmental challenges. Ethical concerns include the welfare and sourcing of animals, while environmental issues stem from the generation of biological waste and the resources required for their use and disposal [11–13]. The increasing integration of ultrasound into curricula highlights the need for high-quality, realistic training models that reduce reliance on animal tissues. Ultrasound-compatible synthetic materials can simulate human tissues and organs, providing learners with consistent, anatomically accurate, and reusable alternatives [14, 15]. These models enhance skill acquisition by offering realistic tactile and imaging feedback, enabling practitioners to practice safely and effectively in a controlled environment while addressing ethical and environmental concerns.

While technological advances offer high-fidelity simulation manikins and virtual simulations, the use of animals and animal tissues persists in many programs [6, 16]. Ethical considerations regarding animal welfare and sustainability concerns necessitate careful planning and consideration of alternatives [17–19]. Technological advancements can provide viable alternatives to animal use, including virtual reality and synthetic materials, which could offer realistic experiences without ethical concerns. Live tissue training (LTT) is a broad descriptor referring to the educational method of employing live animals as models for simulation [6], and animal tissues are also used in part-task or hybrid (animal tissue and synthetic materials) models [20]. The use of LTT in SBE is often justified by the concept of simulator fidelity, however the relationship between simulator design and fidelity has been contested [21]. Fidelity in SBHPE should reflect functional task alignment and focus on the functional correspondence between the simulator and the applied context [22].

### Global practices in animal use in health professions research and education

**Fig. 1:**
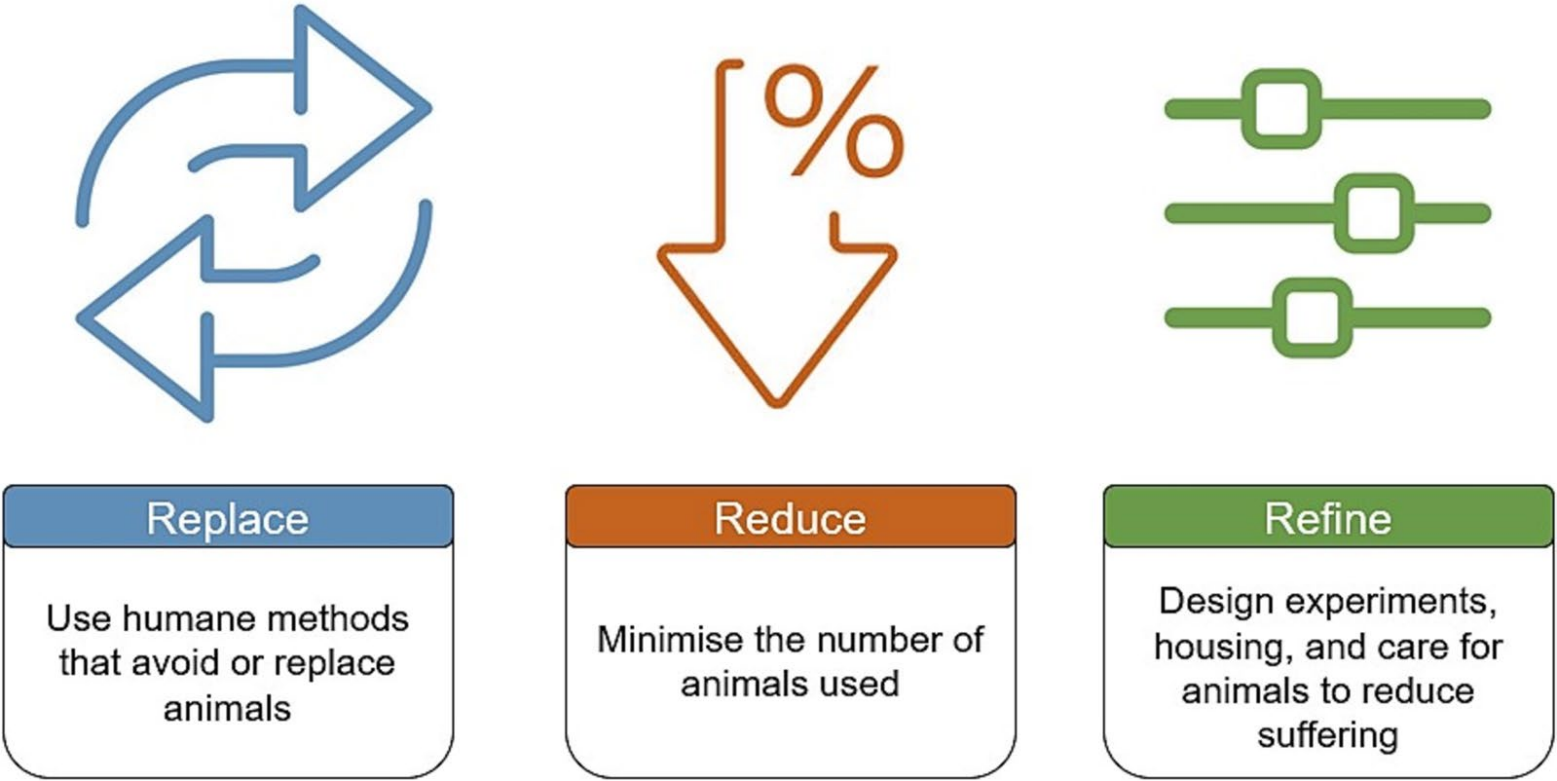
The 3R Model—principles of Replace, Reduce, and Refine

Worldwide it is difficult to estimate the number of animals that are used in medical research and education as international standards differ. However, it is estimated that there are 100 million animals used in research and testing globally per annum, including 12–24 million in the USA, 8.6 million in the EU, and 10.7 million in Australia [23]. In the EU, animals such as rodents, rabbits pigs and cows are used in various educational and training interventions including skills training, surgical training and experiments in physiology [16]. It has been suggested that in any country, 1–10% of animals used in experiments were used in education and training [16], suggesting up to10 million animals may be used in education and training annually. In the UK alone, 50% of the 2.7 million animals used in testing are undertaken in universities and medical schools [23]. To govern the ethical and humane use of animals, countries such as the USA, Japan, and member states in the EU have implemented specific legislation, many utilising the 3Rs model, see Fig. 1.

The principles of Replacement, Reduction, and Refinement offer a comprehensive approach to addressing the ethical and environmental challenges associated with the use of animals in SBE. By adopting non-animal alternatives where possible, simulation educators can reduce the use of animal cadavers, thus reducing the generation of biological waste and disposable waste supplies such as gloves, syringes, and drapes contaminated with animal tissue. Overall this could reduce the waste footprint of SBE programs, and support the United Nations’ Sustainable Development Goals, specifically goals 3, 9,12, and 15 [24]. The modernisation of health professions education has seen an increase in innovation in technology and also an increase in activism in the animal rights movement [17, 25]. Numerous alternatives to the harmful use of animals are accessible, yet the rationale often provided for continuing this practice is that animals must be used for *proper* learning due to the absence of acceptable alternatives [16].

Internationally, health professions schools and accrediting bodies have started to re-evaluate the use of LTT and animal tissue, and have introduced regulations and guidelines for non-animal training. These organisations include the Medical, Pharmacy and Dental Councils of India, as well as the American Emergency Nurses Association, the American Heart Association and the American College of Surgeons [19]. Studies have compared the use of LTT and animal tissue models to synthetic models in health professions and veterinary education and found comparable educational outcomes with no significant difference [5, 26–28]. Advancements in simulation technology have led to the cessation of animal use in 99% of ATLS programs across the United States and Canada, with reported savings of 25–60 USD per student per annuum [29]. However, there is still a preference for animal models in surgical and medical education [6, 7, 30], despite the costs related to the care of animals, including expenses for veterinary staff, supplies, anaesthesia, feeding, and disposal [16]. Crucially, health professions educators must consider if the tissue and anatomical fidelity in animal models are essential to the specific learning objectives of educational interventions?

### Leveraging knowledge and skills from ultrasound quality assurance

Ultrasound simulation-based education (SBE) relies on tissue-mimicking materials (TMMs) that not only replicate the visual and tactile characteristics of clinical tissue but also accurately mimic the specific imaging properties required for ultrasound. Meeting these complex requirements has historically led health profession educators to use animal tissue-based simulators. However, a vast body of research spanning nearly five decades on ultrasound quality assurance (QA) phantoms and TMMs remains underutilized in SBE development [31–33].

QA phantoms are critical in diagnostic imaging, ensuring that equipment used in routine clinical practice maintains optimal performance and that any decline in image quality is correctly identified rather than mistaken for clinical anomalies [33]. Effective QA phantoms must align with clinical applications of ultrasound systems and pre-emptively detect potential issues, thus averting clinical repercussions [33]. Extensive research has characterized and adapted a wide range of commercially available and literature-described test phantoms for various ultrasound QA and image analysis applications [31–34], offering valuable insights for SBE.

An ideal ultrasound test phantom incorporates TMMs with acoustic properties closely resembling those of soft tissue, including speed of sound (SOS), attenuation coefficient, and backscatter [33]. These properties ensure realistic simulation of ultrasound behaviour, providing accurate feedback during scanning and facilitating skill development. Synthetic or non-biological TMMs commonly used in QA phantoms can be engineered with these relevant acoustic characteristics and offer significant advantages. The acoustic characteristics can also be measured to quantitatively match the TMM acoustic characteristics to that of the relevant soft tissues [31]. They are cost-effective, relatively simple to produce, and highly adaptable for various anatomical applications [14, 31, 35, 36]. Additionally, their stability and reproducibility, as documented in the literature, make them particularly suitable for ultrasound SBE, ensuring consistent and reliable training experiences [31, 37]. This synergy between QA research and SBE development presents an opportunity to advance simulation training by leveraging well-characterized and versatile TMMs.

### Collaboration to catalyse change

The expertise, abilities, and perspectives of medical professionals, educators, academic researchers, and industry specialists are essential for developing alternative methods to live animal simulators [7]. Replacing animal tissue in ultrasound training with tissue-mimicking ultrasound-compatible materials marks a significant step forward in creating more ethical and sustainable medical education. Synthetic models provide consistent, reproducible training environments that accurately replicate human anatomy, facilitating precise skill development without the ethical concerns tied to animal use. These models support standardisation in training, ensuring all practitioners receive uniform, high-quality education, while also enabling continuous adaptation to evolve ultrasound technologies and applications.

Transitioning from animal models to synthetic models will require a clear roadmap that includes several key actions. First, there must be a concerted effort to develop and validate tissue-mimicking materials (TMMs) that closely replicate the relevant anatomical and clinical characteristics of human tissues. Quantitative characterisation of these models will be crucial to demonstrate their efficacy in realistic training scenarios. Next, health professions educators and learners must be engaged in evaluating these synthetic models within their curricula to build confidence in their effectiveness for skill development. This can be achieved through pilot programs, workshops, and peer-reviewed studies that compare synthetic models to traditional animal-based methods in terms of training outcomes.

Despite the clear advantages, the transition may face several barriers, including resistance from educators and institutions accustomed to animal models, as well as the perceived high upfront cost of developing and implementing synthetic models. To overcome these challenges, stakeholders must emphasise the long-term benefits of synthetic models, such as cost-effectiveness due to their reusability and ethical and environmental advantages. Additionally, establishing funding opportunities and partnerships with industry leaders in medical simulation can help support the transition. Building a broad consensus within the health professions education community, coupled with strong evidence of the efficacy of synthetic models, will be crucial for reducing reliance on animal models and ensuring the widespread adoption of sustainable alternatives.

## Data Availability

No datasets were generated or analysed during the current study.

## Abbreviations

TMM: Tissue Mimicking Material
3Rs: Replacement, Reduction, and Refinement
SBE: Simulation-Based Education
PoCUS: Point of Care Ultrasound
LTT: Live Tissue Training
QA: Quality Assurance
